# Is *Kiki* angry and *Bouba* happy? Association between emotions, shapes, and sounds

**DOI:** 10.1007/s00426-025-02158-5

**Published:** 2025-07-14

**Authors:** Lari Vainio, Xinyuan Mo, Martti Vainio

**Affiliations:** Department of Digital Humanities, Faculty of Arts,, University of Helsinki, Unioninkatu 38, Helsinki, Finland

## Abstract

Research has shown that particular shapes and speech sounds have common higher-order emotional properties, which might mediate associating angular shapes with kiki-like words and round shapes with bouba-like words, resulting in the so-called kiki-bouba effect. However, research supporting this account has mostly recruited explicit association tests to investigate whether people link particular emotions with these shapes and pseudo-words. This study investigated whether the kiki-bouba effect, observed in the implicit association test, can be similarly based on these emotional mediation processes. We found that the explicit and implicit association tests robustly produced a link between angular shape and angry facial expressions, whereas the round shape was associated with happy and calm facial expressions. In contrast, aurally presented kiki and bouba-like words were associated with these facial expressions in the explicit association test but not in the implicit association test. These observations suggest that people process implicitly the emotional properties of angular/round shapes, while they do not automatically process the emotional properties of the perceived kiki/bouba-like words when the task emphasizes implicit association processes. Consequently, we propose that the kiki-bouba effect, which is observed in explicit association tests, can be partially based on emotional mediation processes. In contrast, the kiki-bouba effect, which is based on implicitly operating association processes, is not likely to be based on emotional mediation processes.

## Introduction

People tend to naturally associate two things, objects, and/or attributes even when they are not self-evidently related to each other. Perhaps, the most common example of this phenomenon is so-called kiki-bouba effect (also known as takete-maluma effect) in which particular speech sounds are associated with particular shapes (Köhler, 1929). Specific vowels (e.g.,/i/) and consonants (e.g.,/t/and/k/) are associated with angular shapes, while some other vowels (e.g.,/o/) and consonants (e.g.,/m/and/b/) are associated with round shapes (Maurer et al., 2006), given that consonants seem to play a more important role than vowels in the effect (Nielsen & Rendall, 2011; Fort et al., 2015).

Other famous examples of such relatively systematic associations– also relevant for the present study– that people make are between shapes and emotions (shape-emotion correspondence) and between sounds and emotions (sound-emotion correspondence). Shape defines how pleasing or displeasing visual stimuli are experienced to be. In general, round/curved shapes are more pleasing than angular/sharp-edged shapes (Bar & Neta, 2006). Regarding sound-emotion associations, particular acoustic features of speech sounds (e.g., perceived pitch and intensity) (Banse & Scherer, 1996) as well as acoustic features of artificial sounds (e.g., the spectral centroid; SC) (Sievers et al., 2019) are systematically associated with different emotions: For example, sounds with higher perceived pitch and intensity as well as mean SC are associated with high-arousal emotions such as excitement. Moreover, it has been shown that speech sounds articulated in the front of the mouth are emotionally preferred over those articulated in the back of the mouth (e.g., Körner & Rummer, 2022; Maschmann et al., 2020).

The kiki-bouba effect belongs to the family of sound symbolism effects that demonstrate a systematic relation between the sound of a word and its meaning (Blasi et al., 2016; Sidhu & Pexman, 2018). The three most commonly proposed explanations of the kiki-bouba effect, and many other sound symbolism effects, designate different weights to possible articulatory, acoustic, and emotional processes behind the effect. First, the articulatory accounts (Ramachandran & Hubbard, 2001; Imai et al., 2025) emphasize that people learn to associate, for example, round shapes with bouba-like words since the mouth makes rounded shapes when producing round vowels of these words. Correspondingly, angular shapes are learned to be associated with kiki-like words since the sharp inflection of the tongue on the palate when articulating kiki-like words aligns with the sharp edges of angular shapes. According to Oberman and Ramachandran (2008), the shape-sound association is based on articulatory-acoustic imitation of angular/round shapes, requiring the co-joint activation between auditory sensory representations and motor representation of movements needed to pronounce the sounds. Second, acoustic theories suggest that the sounds produced by sharp or round objects—such as when they bounce on a surface—contain distinct features that people learn to associate with the corresponding shapes (Fort & Schwartz, 2022; Passi & Arun, 2024; Silva & Bellini-Leite, 2020). Presumably, this account assumes that the acoustic features of, for example, the consonants [t] and [k] have fundamental similarities with the acoustic features of the sound that spiky objects produce, and consequently, people learn to associate these speech sounds with the spiky shapes. If the articulatory accounts typically acknowledge that, in addition to articulatory properties, the acoustic consequences of particular articulatory gestures also have an important role in the kiki-bouba effect (Ramachandran & Hubbard, 2001), the acoustic accounts typically assume that the only relevant speech-related factors in the kiki-bouba effect are exclusively linked to acoustic features of pronounced words (Passi & Arun, 2024). Finally, the emotional model that is proposed to explain the kiki-bouba effect is in the focus of this paper. In short, emotional models assume that angular/round shapes and kiki/bouba-like words have common higher-order emotional properties that in turn mediate the effect (Aryani et al., 2020).

### Are emotional factors mediating the kiki-bouba effect?

Research on emotional sound symbolism assumes that individual phonemes can be statistically associated with negative and positive emotions (Adelman et al., 2018). Emotional connotations linked to different colors can influence the recognition of emotional words, and to some extent also pseudowords (Bortolotti et al., 2024; Garrido et al.,^2019^). As an example of emotional sound symbolism interjections expressing pain across languages worldwide are largely characterized by a preference for/a/-like vowels (Ponsonnet et al., 2024). In fact, research and debate about on emotional mediation of various sound symbolism phenomena is extensive (Spence & Di Stefano, 2022, 2024; Bortolotti et al., 2025). In line with the emotional accounts of the kiki-bouba effect, research shows that when people are asked to judge arousal values of angular and round shapes as well as kiki- and bouba-like words, angular shapes and kiki-like words are rated more aroused than round shapes and bouba-like words along the axis between very calm and very excited (Aryani & Jacobs, 2018; Aryani et al., 2020). The view assumes that this common higher-order emotional property mediates associating a particular shape with particular speech sounds.

It is important to emphasize that the emotional mediation account of the kiki-bouba effect is primarily based on research showing that the angular/round shapes and kiki/bouba-like words are experienced as arousing/calming, respectively (Aryani et al., 2020). However, the fact that two things share the same emotional connotations does not necessarily indicate that these shared higher-order emotional properties provide a fundamental and causal factor that connects these two things and consequently produces the effect. The shape-sound association can be exclusively based on, for example, articulatory or acoustic processes, even when they share the same emotional connotations. However, Aryani et al. (2020; Experiment 3) presented a study, which suggests that evaluated arousal of kiki/bouba-like pseudo-words predicts whether the word is associated with an angular or round shape, which in turn supports the view that associating kiki and bouba-like words with particular shapes can be indeed at least partially mediated by emotional factors.

For the kiki-bouba effect to be considered based on emotional mediation processes, the shapes and pseudo-words involved must be equally associated with corresponding emotions. The previous research has provided support for this view using tasks in which participants are asked to make explicit evaluations of emotional aspects of the kiki/bouba-like words and angular/round shapes (Aryani et al., 2020). In addition to further exploring these associations between kiki/bouba words and emotions as well as angular/round shapes and emotions using an explicit association test, the present study also recruits a more implicit association test to explore this prerequisite for accepting the emotional mediation account of the kiki-bouba effect. Hence, this study explores whether the common higher-order emotional properties of shapes and pseudo-words can be revealed using the implicit association test instead of solely asking participants to explicitly evaluate, for example, the arousal of the stimulus.

The study uses a modified version of the implicit association test (IAT) reported by Parise and Spence (2012; see also Chen & Huang, 2023). Greenwald et al. (1998) originally reported this method. On contrary to implicit responses, explicit responses are highly intended and controllable and include high-level cognitive processes and conscious decision-making (Nosek, 2007). IAT effects have been proposed to reflect associations between concepts in semantic memory (e.g., Greenwald & Nosek, 2001). However, it has been also proposed that despite having the term ”implicit” in its name, in some instances IAT effect might also reflect the outcome of explicit cognitive processes (Blair, 2002; Fiedler et al., 2006). Nevertheless, it can be stated that there are fundamental differences between cognitive mechanisms required for observing association effects in the explicit and implicit association tests. It has been shown that implicit and explicit processes can operate in parallel. Relevantly for the present study, associative processes of cognition, observed for example in semantic priming, can be based on implicit (or automatic) and explicit (or controlled) processes, and the involvement of each of these processes in the effect depends on, for example, the task demands (e.g., Hill et al., 2002). Regarding the kiki-bouba effect, for instance, when participants are asked to explicitly bring together angular/round shapes and kiki/bouba-like words, they are typically given plenty of– sometimes even limitless– time to cogitate the ” smoothness” and ”sharpness” of kiki- and bouba-like words. They are required to deliberately and intentionally ponder whether kiki- or bouba-like word ”feels” more angular or round. In contrast, in implicit association tasks such as IAT, participants are required to respond as fast as possible. The responses can be performed even within 600 ms after the onset of the stimulus when the stimuli are relatively simple, such as a shape (e.g., Parise & Spence, 2012). In implicit tasks, participants are not encouraged to ponder, for example, a perceptual shape of kiki/bouba-like words. Instead, they can be, for example, simply asked to associate a particular shape and a particular pseudoword with a specific response key and respond as fast as possible when they perceive one of these stimuli. It is assumed that responses are performed faster when semantic memory processes support grouping together the two stimulus dimensions, such as shape and pseudoword.

For the sake of providing an example of IAT, in Parise and Spence’s audio-visual version of the IAT, the participants were presented with stimuli (kiki- and bouba-like words as well as angular and round shapes) from which some were hypothesized to be congruent (the kiki-like word and the angular shape as well as the bouba-like word and the round shape) while others were hypothesized to be incongruent (the kiki-like word and the round shape as well as the bouba-like word and the angular shape). The participants were asked to categorize stimuli by pressing one of the two response keys according to the identity of the stimulus (e.g., press the right key if the word is [tɑkete] and if the shape is angular, whereas press the left key if the word is [mɑbemɑ] and if the shape is round). In IAT tasks, the congruent and incongruent response-to-stimulus conditions are typically presented in their own blocks. Generally, the IAT effect is based on the idea that participants respond faster when the same response key is used for responding to congruent stimulus items in comparison to incongruent stimulus items. Indeed, regarding the study of Parise and Spence, responses were significantly faster when one response key was assigned to kiki-like words and angular shapes and another response key was assigned to bouba-like words and round shapes (i.e., congruent response-to-stimulus conditions) in comparison to incongruent response-to-stimulus conditions.

The other primary difference between the present study and the previous studies that have provided evidence for the emotional account of the kiki-bouba effect (Aryani & Jacobs, 2018; Aryani et al., 2020) is that, unlike those previous investigations, this study investigates whether valence-related processes can also account for the kiki-bouba effect in addition to exploring the mediating role of arousal in the effect. In fact, when shape-emotion associations are investigated outside of the context of kiki-bouba phenomenon, instead of emphasizing how the arousal dimension is experienced in different shapes, round shapes are typically experienced as more pleasurable than angular shapes (see the review by Chuquichambi et al., 2022) suggesting that if an emotional factor mediates the kiki-bouba effect, the mediating factor is likely to be even more based on valence than arousal. As such, this study aims to provide further evidence for the emotional account of the kiki-bouba effect by clarifying the emotional mechanisms that might underlie the effect.

It has been recognized that different emotions typically have different degrees of valence and arousal (Russell & Barrett, 1999). Regarding these two emotional dimensions, calmness has a very low degree of excitement (i.e., arousal) and a relatively high degree of pleasantness, happiness has a relatively high degree of excitement and a very high degree of pleasantness, anger has a very high degree of excitement as well as unpleasantness, and sadness has a relatively low degree of excitement and a very high degree of unpleasantness. Hence, we can assume that if, for example, the sharp shape is exclusively associated with a high degree of excitement instead of being positively or negatively associated with valence, it should be linked to anger and happiness rather than calmness and sadness. In contrast, if the sharp shape is also associated with, for example, a high degree of unpleasantness, it should be linked to anger and sadness rather than calmness and happiness. Therefore, this study uses various images of facial expressions that present these four emotions: calmness, happiness, anger, and sadness. The facial stimuli consist partly of the emotional faces of real people and partly of the emotional facial emoji symbols. In addition to using these emotional faces as stimuli in the IAT study, we also verify the arousal and valence values of these stimuli by asking participants to explicitly judge their valence and arousal rates. We hope that this procedure will help us to clarify to what degree arousal and/or valence of the stimuli is responsible for the kiki-bouba effect, hypothetically observed in the study.

### Objectives of the study regarding the shape-emotion and sound-emotion correspondences

This study also aims to contribute, at the general level, to the understanding of shape-emotion and sound-emotion correspondence phenomena. As already mentioned, regarding shape-emotion correspondence, when this phenomenon has been explored outside of the context of the kiki-bouba effect, research either focuses on the association between shapes (round/angular) and valence (pleasurable/unpleasurable) or does not clearly specify whether the underlying emotion is based on arousal or valence (Bar & Neta, 2006; Chuquichambi et al., 2022). Therefore, it is not entirely clear whether, and to what extent, the shape-emotion correspondence is based on experienced valence or arousal of the perceived shapes. In addition, the studies investigating the shape-emotion correspondence have nearly exclusively used explicit evaluation tasks in which participants are asked to judge, for example, the emotional preference of different shapes. We managed to find only one study that has shown the shape-emotion correspondence effect using an implicit association task (Palumbo et al., 2015). Therefore, we aim to provide additional evidence for the shape-emotion correspondence in the context of implicit processing.

In addition, when the sound-emotion correspondence phenomenon has been explored outside of the context of the kiki-bouba effect, the phenomenon has mostly focused on measuring the experienced valence of perceived speech sounds or pseudo-words. Shortly, speech sounds that are articulated in the front of the mouth such as the front vowel [i] and the bilabial consonant [m] are emotionally preferred over those articulated in the back of the mouth such as the back vowel [o] and the velar stop [k] (Maschmann et al., 2020; Körner & Rummer, 2022). As such, although in the context of the kiki-bouba effect, the sound-meaning correspondence has been linked to the experienced arousal of the perceived pseudo-word (Aryani & Jacobs, 2018; Aryani et al., 2020), outside of this research context, the sound-emotion correspondence has been typically associated with the experienced valence of the pseudo-word. In addition, the studies investigating the sound-emotion correspondence have nearly exclusively used explicit evaluation tasks in which participants are asked to judge, for example, the emotional preference of different pseudo-words. We found only one study investigating sound-emotion correspondence in the context of an implicit association task (Schmidtke et al., 2024). Hence, taken together, in addition to exploring underlying mechanisms behind the kiki-bouba effect, this study can also provide new knowledge for understanding the emotional processes behind shape-emotion correspondence as well as sound-emotion correspondence.

## Experiment 1

Experiment 1 aims to reveal the kiki-bouba effect between audible pseudo-words ([tɑketɑ] and [mɑbemɑ]) and visual shapes (angular and round) using the IAT. We only manipulated the consonant content between the words because it has been shown that consonants play a more important role than vowels in the effect (Nielsen & Rendall, 2011; Fort et al., 2015). The consonants [t] and [k] were selected for the kiki-like word and the [m] and [b] for the bouba-like words because the [t] and [k] are associated very strongly with sharp shapes whereas the [m] and [b] are associated very strongly with round shapes (Fort et al., 2015). The shape stimuli used in the study were similar to those that have been previously used in many ”kiki-bouba” studies (e.g., Bremner et al., 2013; Chen et al., 2016). In addition, Experiment 1 aims to reveal the shape-emotion and sound-emotion correspondences concerning the arousal of the emotional facial stimuli. Therefore, the emotional stimuli consisted of angry and calm facial expressions. In addition to using images of real faces in the emotional stimuli, we also used emoji faces because angry facial expressions might contain more angular features than calm facial expressions (Watier, 2018), which could create a connection between angry faces and angular shapes, even without the influence of emotional processing of the stimuli. The emoji faces were constructed so that the angry and calm expressions contained identical round and angular features.

Finally, in addition to measuring how particular shapes, sounds, and emotions are implicitly associated with each other, we also measure how the arousal and valence dimensions are perceived in each stimulus by asking the participants to evaluate these dimensions for the stimuli. In this way, we can determine the experienced valence and arousal values of all stimuli and evaluate whether the potential effects observed in the IAT are more likely to be based on the arousal or valence of the stimuli. Moreover, it was assumed that if the kiki-bouba effect, which is potentially observed in the present IAT task, is based on emotional mediation processes, a similar IAT effect should be observed between shapes and emotions as well as pseudo-words and emotions associating the angry emotional expressions with the sharp shape and the word [tɑketɑ] and the calm emotional expressions with the round shape and the word [mɑbemɑ]. In addition, this emotional mediation hypothesis would be supported by observing similar valence and/or arousal rates for the sharp shape and the [tɑketɑ] word as well as the round shape and the [mɑbemɑ] word in the explicit emotion evaluation task.

### Method

#### Participants

In Experiment 1, eighteen people participated in the study (19–34 years of age; mean age = 25.6 years; 5 males; all right-handed). All participants had normal or corrected-to-normal vision and hearing and were native Finnish speakers. All participants were naive to the purpose of the study. The informed consent to participate in the study was obtained from participants. None of the participants took part in more than one of the four experiments in this study. Given that the study was a modified version of the implicit association test (IAT) reported by Parise and Spence (2012), and ten participants were recruited for that study providing a significance level of *p* <.001, we presumed that eighteen participants would suffice to achieve an appropriate statistical power. In addition, we carried out the power simulations based on our earlier dataset from an experiment (Vainio et al., 2025: Experiment 2) with a similar IAT design (see https://osf.io/5eqkg/?view_only=19eee5cb010f42479a30e2c06844c664 for the simulated data [IAT.sav] and the R script that was used to simulate the data). The participants (*n* = 19) had a random effect on the intercept and the slope of congruency. The simulations suggest that already 12 participants would suffice to produce a statistically significant difference in 96% of experiments. The simulations were run with R package simr (Green & MacLeod, 2016). The sample size calculation naturally assumes that the effect observed in the present study is roughly of the same magnitude as observed in the previous study. Therefore, we used six more participants than the minimum suggested by the power simulations. The study was conducted according to the principles expressed in the Declaration of Helsinki. Written informed consent was obtained from all participants, and the participants were able to withdraw their consent and participation at any time without consequence. The participants received gift cards valued at 20 euros as compensation for participating. The study was approved by the Ethical Review Board in the Humanities and Social and Behavioral Sciences at the University of Helsinki.

#### Apparatus, stimuli, procedure, and statistical analyses

Each participant sat in a dimly lit room with his or her head 80 cm in front of a 25-inch Full HD monitor (screen refresh rate: 60 Hz; screen resolution: 2560 × 1440). Presentation^®^ software (V16.1; https://www.neurobs.com) was used to execute the study. Responses were executed with the Cedrus response pad, which was located at the front of the monitor.

**Fig. 1:**
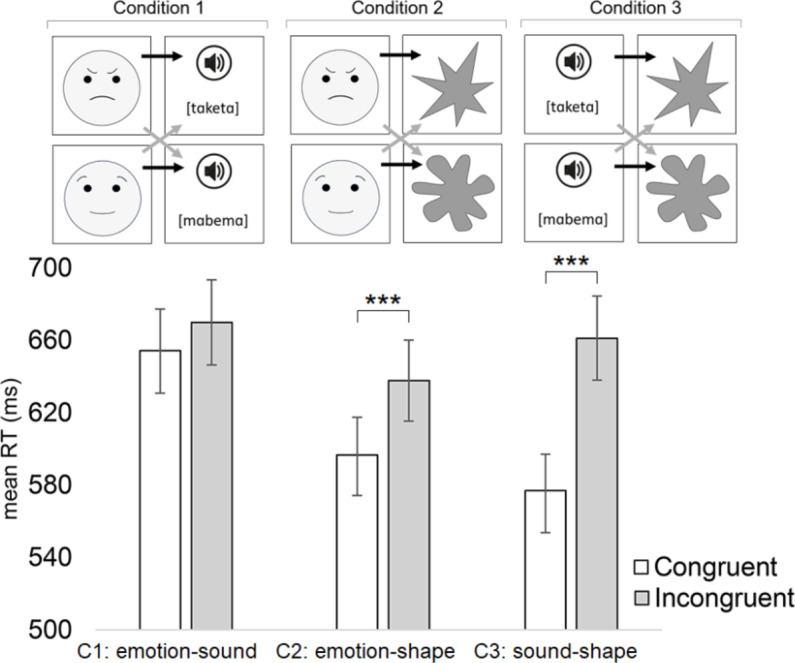
(Above) The illustration of the stimuli and design used in Experiment 1. The black arrows refer to congruent stimulus pairs whereas grey arrows refer to incongruent stimulus pairs. Notice that Experiments 1 and 2 also included images of real facial expressions. Original material showing these real facial expressions stems from MPI FACES database (persons #139 [angry_a, happy_a, sad_a] and #171 [angry_a, happy_a, sad_b, neutral_b], Ebner et al., 2010). Depiction used with kind permission of the Max Planck Institute— further distribution, publication, or display beyond illustrating the research methodology of this study is prohibited by the Max Planck Institute. In Conditions 1 and 2 of Experiment 1, stimuli consisted of real facial expressions (25%), corresponding facial expressions of emoji stimuli (25%), and [tɑketɑ]/[mɑbemɑ] stimuli (50% in Condition 1), or angular/round shapes (50% in Condition 2). (Below) The mean manual reaction times for Experiment 1 as a function of the congruency for the conditions of emotion-word, emotion-shape, and shape-word. In the emotion-word condition, the congruent pairs were angry-[tɑketɑ] and calm-[mɑbemɑ], in the emotion-shape condition, the congruent pairs were angry-angular and calm-round, and in the shape-word condition, the congruent pairs were angular-[tɑketɑ] and round-[mɑbemɑ]. Error bars depict the standard error of the mean. Asterisks indicate statistically significant differences (***p <.001)

The auditory stimuli consisted of words [tɑketɑ] (404 ms) and [mɑbemɑ] (403 ms) vocalized in a female voice. Praat software was used to make the intensity and fundamental frequency (217 Hz) identical for both auditory stimuli. We avoided including those consonants in these pseudo-words that are included in the Finnish words of angry (’vihainen’), happy (’iloinen’), sad (’surullinen’), and calm (’levollinen’). Each auditory stimulus was presented over headphones (at ca. 68 dB SPL). The visual stimuli consisted of angular and round shapes (see Fig. 1) that were presented in two sizes (smaller angular/round: horizontal 6.1 cm; vertical 6.8 cm; larger angular/round: horizontal 8.6 cm; vertical 9.4 cm) in order to avoid participants using size as a response cue (notice that the round shape looks a bit larger because it has thicker ”limbs” even though it has the same horizontal and vertical lengths as the angular shape). The visual stimuli also consisted of images of a female’s angry and calm facial expressions and emoji face versions of these facial expressions (real face: horizontal 8.5 cm; vertical 9.6 cm; emoji face: horizontal 9.2 cm; vertical 9.2 cm) (see Fig. 1). The real facial expressions were borrowed from MPI FACES database (#171 [angry_a and neutral_b]; Ebner et al., 2010). All visual stimuli were displayed at the center of the monitor and in black-and-white color.

The experiment consisted of twelve experimental blocks (48 trials in each block; 576 experimental trials overall). There was a practice block at the beginning of each actual block consisting of sixteen trials (192 practice trials overall). Each stimulus of the given block was presented two times in each practice block. Half of the blocks were congruent and half were incongruent blocks. In congruent blocks, the same response keys were allocated for the congruent stimuli (e.g., the right key for the [tɑketɑ] and the angular shape and the left key for the [mɑbemɑ] and the round shape), while in incongruent blocks, different response keys were allocated for the congruent stimuli (e.g., the right key for the [tɑketɑ] and the round shape and the left key for the [mɑbemɑ] and the angular shape). There were two versions of congruent and incongruent response key arrangements. That is, for example, in one congruent version, the left key was allocated for the [mɑbemɑ] and the round shape, and the right key was allocated for the [tɑketɑ] and the angular shape. In the alternative version, the left key was allocated for the [tɑketɑ] and the angular shape, and the right key was allocated for the [mɑbemɑ] and the round shape. The order of the blocks was randomized, however, so that two congruent or incongruent versions of the same block were never presented one after another.

A single trial started with a fixation cross that was presented for 1000 ms. Then a blank screen was displayed for 800 ms. Finally, the target stimulus was presented. The target was either one of the visual or auditory stimuli. The visual targets were presented for 1800 ms. All trials waited for responses for 2000 ms. That is, a blank screen was displayed for 2000 ms from the onset of the auditory target and for 200 ms from the offset of the visual target. A response initiated a new trial. Errorneus responses were immediately followed by presenting red ’ERROR’ text for 800 ms.

Responses were performed with the right hand. The left response was performed with the index finger, and the right response was performed with the middle finger. The left response key was indicated by the letter V (the initial letter of the word ’vasen’, which is ’left’ in Finnish), presented above the left response key, and the right response key was indicated by the letter O (the initial letter of the word ’oikea’, which is ’right’ in Finnish). The block instructions were displayed at the beginning of the practice trials of each block. The instructions stated that, for example, the right key (O) should be pressed as fast as possible if the auditory word is [tɑketɑ] or the visual shape is angular, while the left key (V) should be pressed if the auditory word is [mɑbemɑ] or the visual shape is round. Regarding the categorizing of facial expressions, the participants were asked to select the response key depending on whether the facial expression was angry or calm.

At the end of the study, participants answered the questionnaire, which tested the experienced valence (scale between 1 [very unpleasant] and 7 [very pleasant]) and arousal (scale between 1 [very calm] and 7 [very excited]) of the face, shape and word stimuli used in the study. This questionnaire also tested whether participants associated the word [tɑketɑ] and [mɑbemɑ] with the angular or round shape (1 = very angular, 2 = quite angular, 3 = mildly angular, 4 = mildly round, 5 = quite round, 6 = very round). It was also asked whether the participant thinks that the angry and calm face is associated with the word [tɑketɑ] or [mɑbemɑ] (1 = very [tɑketɑ], 2 = quite [tɑketɑ], 3 = mildly [tɑketɑ] 4 = mildly [mɑbemɑ], 5 = quite [mɑbemɑ], 6 = very [mɑbemɑ]), and whether these facial expressions are associated with angular or round shape (1 = very angular, 2 = quite angular, 3 = mildly angular, 4 = mildly round, 5 = quite round, 6 = very round). Finally, it was asked whether the participant was familiar (yes/no) with the kiki-bouba effect in which particular types of words such as [tɑketɑ] are associated with angular shapes, while some other types of words such as [mɑbemɑ] are associated with round shapes.

The statistical significance of observed differences in the IAT was assessed using the generalized linear mixed model (GLMM) analysis framework by implementing a gamma distribution assumption (log link function) for the dependent variable of reaction time (RT). In the analysis of accuracy, the dependent variable was the percentage of erroneous responses. Regarding independent variables, the GLMM analyses treated block Condition (1 = [angry^a^/calm^b^] emotion– [tɑketɑ^c^/mɑbemɑ^d^] word, 2 = [angry^a^/calm^b^] emotion– [angular^c^/round^d^] shape, 3 = [angular^a^/round^b^] shape - [tɑketɑ^c^/mɑbemɑ^d^] word) and block Congruency (1 = congruent block, 2 = incongruent block) as fixed within factors. Note that in each block condition, the item marked with a superscript ‘a’ is hypothesized to be congruent with the item marked with a superscript ‘c’, while the item marked with a superscript ‘b’ is hypothesized to be congruent with the item marked with a superscript ‘d’. In addition, there was a random intercept of Subject with a random slope of Congruency and Condition. Pairwise comparisons were adjusted using Bonferroni correction. All analyses were conducted using the SPSS statistics software package (version 28).

### Results

*Reaction times*: Before analyzing reaction times, all practice trials and erroneous data (i.e., the participant responded with a wrong key or did not produce any response; 5.1%) were removed. Additionally, reaction time data more than 2 standard deviations from the mean (performed individually for the data of each participant) were excluded (4.9%). The data of one participant was removed because 13.8% of her responses were incorrect. The analysis included the within-subjects variables of Condition (1 = emotion-word, 2 = emotion-shape, 3 = shape-word) and Congruency (1 = congruent block, 2 = incongruent block). The reaction time analysis revealed significant main effects of Condition [F(2, 8823) = 33.81, *p* <.001] (Condition 1: M = 662 ms; Condition 2: M = 617 ms; Condition 3 = 618 ms) and Congruency [F(1, 8823) = 61.83, *p* <.001] (Congruent: M = 608 ms; Incongruent: M = 656 ms). In addition, the interaction between Condition and Congruency was significant [F(2, 8823) = 39.09, *p* <.001]. A statistically significant congruency effect was observed in Conditions 2 and 3 (Condition 1: Congruent: M = 654 ms, Incongruent: M = 670 ms, *p* =.052, *d*_*z*_ *=* 0.17; Condition 2: Congruent: M = 597 ms, Incongruent: M = 638 ms, *p* <.001, *d*_*z*_ *=* 0.46; Condition 3: Congruent: M = 577 ms, Incongruent: M = 661 ms, *p* <.001, *d*_*z*_ *=* 0.94). These interactions are provided in Fig. 1.

In order to test whether the congruency effect differs between real facial stimuli and emoji stimuli in Conditions 1 and 2, we conducted separate analyses including either only the real facial stimuli or only the emoji stimuli in each analysis. The analysis of real facial stimuli revealed a significant interaction between Condition and Congruency [F(1, 4447) = 13.09, *p* <.001]. The congruency effect was only observed in Condition 2 (Condition 1: Congruent: M = 658 ms, Incongruent: M = 664 ms, *p* =.581, *d*_*z*_ *=* 0.06; Condition 2: Congruent: M = 590 ms, Incongruent: M = 627 ms, *p* <.001, *d*_*z*_ *=* 0.42). The analysis of emoji stimuli similarly revealed a significant interaction between Condition and Congruency [F(1, 4434) = 9.80, *p* =.002]. The congruency effect was only observed in Condition 2 (Condition 1: Congruent: M = 646 ms, Incongruent: M = 662 ms, *p* =.091, *d*_*z*_ *=* 0.16; Condition 2: Congruent: M = 579 ms, Incongruent: M = 622 ms, *p* <.001, *d*_*z*_ *=* 0.48).

In order to test whether the participants’ familiarity with the kiki-bouba effect influenced the congruency effect, we performed an analysis for the data of Condition 3 so that the familiarity was added as a new variable (1 = unfamiliar, 2 = familiar). The analysis revealed a significant interaction between Congruency and Familiarity [F(1, 2918) = 4.68, *p* =.031]. Although the congruence effect was observed for both participant groups, the congruency effect was larger for the participants who knew the effect (Congruent: M = 598 ms, Incongruent: M = 707 ms, *p* <.001, *d*_*z*_ *=* 0.85) than for those who did not know the effect (Congruent: M = 558 ms, Incongruent: 622 ms, *p* <.001, *d*_*z*_ *=* 0.58).

Finally, the results of erroneous responses revealed a significant difference between congruent and incongruent responses [F(1, 87) = 29.46, *p* <.001] (congruent: M = 2.8%; incongruent: M = 5.3%; *d*_*z*_ = 1.1). The interaction between Congruency and Condition was also significant [F(2, 87) = 3.97, *p* =.022] showing that participants made significantly fewer congruent errors in Conditions 2 (*p* =.015; congruent: M = 2.7%; incongruent: M = 4.5%; *d*_*z*_ = 0.65) and 3 (*p* <.001; congruent: M = 2.4%; incongruent: M = 7.8%; *d*_*z*_ = 1.4). The difference between Congruent and Incongruent responses was not significant in Condition 1 (*p* =.284).

#### Questionnaire analysis

The data were first analyzed for the responses that signaled whether a participant associated the angry (1) or calm (2) facial expression with [tɑketɑ] or [mɑbemɑ] sound (1 = very [tɑketɑ], 2 = quite [tɑketɑ], 3 = mildly [tɑketɑ], 4 = mildly [mɑbemɑ], 5 = quite [mɑbemɑ], 6 = very [mɑbemɑ]), and whether a participant associated the angry or calm facial expression with angular or round shape (1 = very angular, 2 = quite angular, 3 = mildly angular, 4 = mildly round, 5 = quite round, 6 = very round). In this analysis, we used the Related-Samples Wilcoxon Signed Rank Test because the data were not normally distributed. The test revealed that the [tɑketɑ]/[mɑbemɑ] rating significantly differed between the two expressions [z(16) = 3.4, *p* <.001, *r* =.8]. The angry expression was associated more strongly with the [tɑketɑ] (M = 2.4, SD = 0.8), while the calm expression was associated more strongly with the [mɑbemɑ] (M = 4.6, SD = 0.9). In addition, the shape rating significantly differed between the two expressions [z(16) = 3.6, *p* <.001, *r* =.9]. The angry expression was associated more strongly with the angular shape (M = 1.6, SD = 0.9), while the calm expression was associated more strongly with the round shape (M = 4.9, SD = 0.7).

The same analysis was carried out to explore the standard kiki-bouba effect; that is, whether participants associate [tɑketɑ] and [mɑbemɑ] sounds with angular or round shapes. The response scale was between 1 and 6 (1 = very angular, 2 = quite angular, 3 = mildly angular, 4 = mildly round, 5 = quite round, 6 = very round). The sound (1 = [tɑketɑ], 2 = [mɑbemɑ]) was the independent variable. The analysis was executed for those who did not know the kiki-bouba effect (1) and knew the effect (2). Concerning those who did not know the effect, the test revealed that the sharpness/roundness rating significantly differed between the two sounds [z(7) = 2.5, *p* =.011, *r* =.9]. The [tɑketɑ] was judged sharper (M = 1.4, SD = 0.5) than the [mɑbemɑ] (M = 5.1, SD = 0.8). Concerning those who know the effect, the test also revealed that the sharpness/roundness rating significantly differed between the two sounds [z(8) = 2.7, *p* =.007, *r* =.9]. The [tɑketɑ] was judged sharper (M = 1.6, SD = 0.7) than the [mɑbemɑ] (M = 5.6, SD = 0.5).

Finally, we recruited Related-Samples Friedman’s Two-Way Analysis of Variance test to analyze valence (between very unpleasant [1] and very pleasant [7]) and arousal (very calm [1] and very excited [7]) ratings between angry-emoji (1), angry-real (2), calm-emoji (3), calm-real (4), angular shape (5), round shape (6), [tɑketɑ] sound (7), and [mɑbemɑ] sound (8). Concerning valence ratings, the analysis revealed a significant difference in ratings between the variables [CHI^2^(7) = 72.7, *p* <.001]. Table 1 shows the means and SDs for each variable and which variables significantly differ from each other according to ratings. Concerning arousal ratings, the analysis revealed a significant difference in ratings between the variables [CHI^2^(7) = 89.5, *p* <.001].

**Table 1:** The tables present the mean rates and standard deviations of the stimuli used in experiment 1. The subscript ’em’ signals to the ’emoji’ version of the stimuli, and the ’re’ signals to the ’real’ version of the stimuli. The upper table presents the estimated Valence rates, while the lower table presents the estimated arousal rates. The tables also present the outcome of Related-Samples friedman’s Two-Way analysis of variance test. The significant p-values between the evaluations of two stimuli signal that the evaluations differ significantly from each other (ns = not significant)

The estimated valence of the stimuli								
	mean/SD	angry^re^	calm^em^	calm^re^	angular	round	[tɑketɑ]	[mɑbemɑ]
angry^em^	2.6/1.0	ns	< 0.001	< 0.001	ns	< 0.001	ns	< 0.001
angry^re^	2.7/1.2		< 0.001	< 0.001	ns	= 0.002	ns	< 0.001
calm^em^	5.8/0.6			ns	< 0.001	ns	= 0.028	ns
calm_re_	5.1/1.2				ns	ns	ns	ns
angular	3.5/1.2					ns	ns	ns
round	5.1/1.3						ns	ns
[tɑketɑ]	4.2/1.3							ns
[mɑbemɑ]	5.1/0.9							
The estimated arousal of the stimuli								
	mean/SD	angry^re^	calm^em^	calm^re^	angular	round	[tɑketɑ]	[mɑbemɑ]
angry^em^	5.2/1.3	ns	< 0.001	< 0.001	ns	= 0.004	ns	< 0.001
angry^re^	5.2/1.1		< 0.001	< 001	ns	= 0.003	ns	< 0.001
calm^em^	1.4/0.5			ns	< 0.001	ns	< 0.001	ns
calm^re^	1.7/0.8				< 0.001	ns	= 0.003	ns
angular	5.1/1.2					= 0.003	ns	< 0.001
round	2.4/0.8						ns	ns
[tɑketɑ]	4.2/1.0							ns
[mɑbemɑ]	2.2/0.9							

### Discussion

The results of Experiment 1 showed the kiki-bouba effect in the explicit as well as implicit association tests. In addition, the study showed that in the IAT, the sharp shape is associated with the angry expression, whereas the round shape is associated with the calm expression. These effects were highly significant, and the effect sizes signaled that the effects were medium or large, suggesting that these interactions are relatively robust. Regarding whether the kiki-bouba effect is more based on arousal or valence dimension of the shape and sound stimuli, if emotional processes indeed mediate the effect, the explicitly estimated emotion rates suggest that the effect might be more likely based on arousal dimension because the angular shape differed from the round shape as well as the [mɑbemɑ] word exclusively in terms of arousal rates. Correspondingly, it seems that the shape-emotion correspondences observed in the IAT of this study might be more robustly based on an experienced arousal of particular shapes instead of an experienced valence of these shapes. That is because, for example, the emotional evaluations of the calm facial expressions differed from the emotional evaluations of the angular shape in terms of arousal rates but not in terms of valence rates. However, the IAT does not conclusively support the view that the implicit kiki-bouba effect would be based on emotional mediation processes. That is because the IAT produced the implicit association effect only between shapes and emotions, while the effect was missing between pseudowords and emotions. The prerequisite for accepting that the kiki-bouba effect can be based on emotional mediation processes was not accomplished, at least concerning the implicit kiki-bouba effect. The shapes and the pseudo-words that were integrated in the kiki-bouba effect were not implicitly associated in the same way with corresponding emotions. This view is rationalized in better detail in the General Discussion.

## Experiment 2

Experiment 2 focuses on further exploring how particular emotions are associated with angular and round shapes. The primary goal of this study was to recruit the IAT to investigate whether sharp and round shapes are associated with high/low arousal and/or valence values. We assumed that this investigation would provide further information about whether and how the the kiki-bouba effect observed in Experiment 1 could be based on emotional processes, and whether the shape-emotion correspondence is more linked to arousal or valence. Again, we used images of real facial expressions as well as emotional facial emoji symbols in which angular/round features were fully controlled. The facial stimuli provided the emotions of happiness, sadness, and anger. Above, we justify why selecting these expressions is optimally suitable for investigating the research questions of the present study. If the shape-emotion correspondence is exclusively based on the arousal of the stimuli, we hypothesize that (1) in the anger-sadness blocks, the angular shape is associated with anger and the round shape is associated with sadness, and (2) in the happiness-sadness blocks, the angular shape is associated with happiness and the round shape is associated with the sadness. In contrast, if the shape-emotion correspondence is based on the valence of the stimuli, we hypothesize that (1) in the anger-happiness blocks, the angular shape is associated with anger and the round shape is associated with happiness, and (2) in the happiness-sadness blocks, the angular shape is associated with sadness and the round shape is associated with the happiness.

### Method

#### Participants

In Experiment 2, twenty-six people participated in the study (20–38 years of age; mean age = 27.1 years; all were females and right-handed). All participants had normal or corrected-to-normal vision and hearing and were native Finnish speakers. All participants were naive to the purpose of the study. The informed consent to participate in the study was obtained from participants. None of the participants took part in multiple experiments. The study was conducted according to the principles expressed in the Declaration of Helsinki. Written informed consent was obtained from all participants, and the participants were able to withdraw their consent and participation at any time without consequence. The participants received gift cards valued at 20 euros as compensation for participating. The study was approved by the Ethical Review Board in the Humanities and Social and Behavioral Sciences at the University of Helsinki.

#### Apparatus, stimuli, procedure, and statistical analyses

The apparatus, procedure, and statistical analyses were the same as in Experiment 1. The shape stimuli were also the same as in Experiment 1. However, unlike in Experiment 1, in Experiment 2, the face stimuli consisted of the two females’ angry, happy, and sad expressions and emoji face versions of these facial expressions. Regarding the categorizing of facial expressions, the participants were asked to select the response key depending on whether the facial expression was angry or happy (Condition 1), sad or happy (Condition 2), and angry or sad (Condition 3). Regarding the categorizing of shapes, the participants were asked to select the response key depending on whether the shape was round or angular. The shapes and faces were presented in the black-and-white color and the same size as the corresponding stimuli in Experiment 1. The real faces were borrowed from the FACES database (Ebner et al., 2010). Original material showing these real facial expressions stems from MPI FACES database of person^a^ #139 (angry_a, happy_a, sad_a) and person^b^ #171 (angry_a, happy_a, sad_b). The questionnaire of Experiment 2 tested the experienced valence (scale between 1 [very unpleasant] and 7 [very pleasant]) and arousal (scale between 1 [very calm] and 7 [very excited]) of the facial expressions of the real and emoji faces as well as the shapes used in the study. Finally, regarding independent variables of the IAT data, the GLMM analyses treated block Condition (1 = [angry^a^/happy^b^] emotion - [angular^c^/round^d^] shape, 2 = [sad^a^/happy^b^] emotion– [angular^c^/round^d^] shape, 3 = [angry^a^/sad^b^] emotion– [angular^c^/round^d^] shape) and block Congruency (1 = congruent block, 2 = incongruent block) as fixed within factors. Notice that in each block Condition, the item that is marked by the superscript ’a’ is hypothesized to be congruent with the item that is marked with the superscript ’c’, while the item that is marked by the superscript ’b’ is hypothesized to be congruent with the item that is marked by the superscript ’d’. In the analysis of accuracy, the dependent variable was the percentage of erroneous responses. Finally, Experiment 2 also included the independent (between-subjects) variable of sub-Experiment (1 = real facial stimuli, 2 = emoji facial stimuli). Thirteen participants carried out the sub-Experiment 1 and thirteen carried out the sub-Experiment 2. There was a random intercept of Subject with a random slope of Congruency, Condition, and sub-Experiment. Pairwise comparisons were adjusted using Bonferroni correction. All analyses were conducted using the SPSS statistics software package (version 28).

### Results

**Fig. 2:**
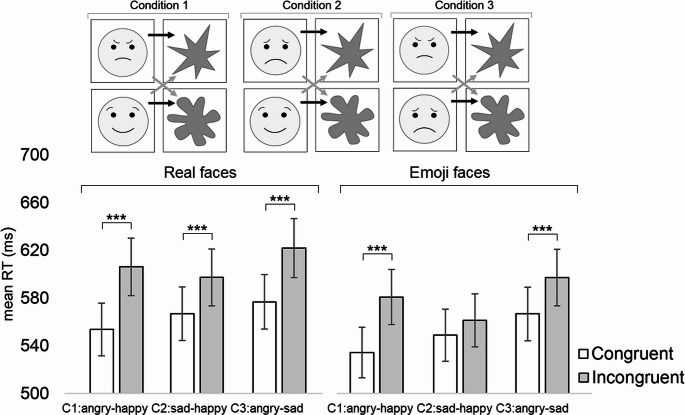
(Above) The illustration of the stimuli and design used in Experiment 2. The black arrows refer to congruent stimulus pairs whereas grey arrows refer to incongruent stimulus pairs. Notice that Experiments 1 and 2 also included images of real facial expressions. Experiment 2 consisted of two sub-experiments from which the first only presented real facial expressions and the second only presented corresponding facial expressions of emoji stimuli. (Below) The mean manual reaction times for Experiment 2, separately for the real faces and emoji faces, as a function of the congruency for the conditions of angry-happy, sad-happy, and angry-sad. In the angry-happy condition, the congruent pairs were angry-angular and happy-round, in the sad-happy condition, the congruent pairs were sad-angular and happy-round, and in the angry-sad condition, the congruent pairs were angry-angular and sad-round. Error bars depict the standard error of the mean. Asterisks indicate statistically significant differences (***p <.001)

*Reaction times*: Before analyzing reaction times, all practice trials and erroneous data (i.e., the participant responded with a wrong key or did not produce any response; 3.9%) were removed. Additionally, reaction time data more than 2 standard deviations from the mean (performed individually for the data of each participant) were excluded (4.3%). The analysis included the within-subjects variables of Condition (1 = angry/happy-angular/round, 2 = sad/happy-angular/round, 3 = angry/sad-angular/round) and Congruency (1 = congruent block, 2 = incongruent block) and the between-subjects factor of sub-Experiment (1 = real facial stimuli, 2 = emoji facial stimuli). The analysis revealed significant main effects of Condition [F(2, 13742) = 23.19, *p* <.001] (Condition 1: M = 567 ms; Condition 2: M = 566 ms; Condition 3 = 591 ms) and Congruency [F(1, 13742) = 48.35, *p* <.001] (Congruent: M = 557 ms; Incongruent: M = 592 ms). In addition, the interaction between Condition and Congruency was significant [F(2, 13742) = 16.15, *p* <.001]. A statistically significant congruency effect was observed in all Conditions (Condition 1: Congruent: M = 542 ms, Incongruent: M = 592 ms, *p* <.001, *d*_*z*_ *=* 0.59; Condition 2: Congruent: M = 556 ms, Incongruent: M = 576 ms, *p* <.001, *d*_*z*_ *=* 0.24; Condition 3: Congruent: M = 573 ms, Incongruent: M = 609 ms, *p* <.001, *d*_*z*_ *=* 0.41). The mean reaction times and pairwise contrasts of this interaction are provided in Fig. 2. The three-way interaction between Condition, Congruency, and sub-Experiment was not significant [F(2, 13742) = 1.39, *p* =.250]. However, the pairwise comparisons test showed that the congruency effect (*p* <.001) was observed in other conditions but not in Condition 2 of sub-Experiment 2 (Congruent: M = 546 ms, Incongruent: M = 555 ms, *p* =.251, *d*_*z*_ *=* 0.08).

In order to test whether the congruency effect differs between the facial stimuli of the person ’a’ and ’b’, we conducted separate analyses for the data from sub-Experiment 1, including only the facial stimuli of person ‘a’ in one analysis and only those of person ‘b’ in the other. The analysis of real facial stimuli of the person ’a’ revealed a significant interaction between Condition and Congruency [F(2, 5245) = 4.05, *p* =.018]. The congruency effect was more robust in Conditions 1 (Congruent: M = 543 ms, Incongruent: M = 596 ms, *p* <.001, *d*_*z*_ = 0.69) and 3 (Congruent: M = 559 ms, Incongruent: M = 599 ms, *p* <.001, *d*_*z*_ = 0.51) in comparison to Condition 2 (Congruent: M = 552 ms, Incongruent: M = 579 ms, *p* =.002, *d*_*z*_ = 0.35). The analysis of real facial stimuli of the person ’b’ revealed a significant interaction between Condition and Congruency [F(2, 5267) = 3.89, *p* =.020]. The congruency effect was more robust in Conditions 1 (Congruent: M = 546 ms, Incongruent: M = 596 ms, *p* <.001, *d*_*z*_ = 0.63) and 3 (Congruent: M = 558 ms, Incongruent: M = 609 ms, *p* <.001, *d*_*z*_ = 0.63) in comparison to Condition 2 (Congruent: M = 558 ms, Incongruent: M = 586 ms, *p* =.002, *d*_*z*_ = 0.35).

The results of erroneous responses revealed significant main effects of sub-Experiment [F(1, 120) = 6.59, *p* =.011] (sub-Experiment 1: M = 2.2%, sub-Experiment 2: M = 3.5%) and Congruency [F(1, 120) = 25.38, *p* <.001] (Congruent: M = 2.2%, Incongruent: M = 3.5%). In addition, there was a significant interaction between Condition and Congruency [F(2, 120) = 6.47, *p* =.002] showing that participants made significantly fewer congruent errors in Conditions 1 (Congruent: M = 1.9%; Incongruent: M = 4.6%; *p* <.001, *d*_*z*_ = 1.10) and 3 (congruent: M = 2.3%; incongruent: M = 3.3%; *p* =.015, *d*_*z*_ = 0.54). The congruency effect was not observed in Condition 2 (Congruent: M = 2.5%; Incongruent: M = 2.7%; *p* =.565, *d*_*z*_ = 0.11). The three-way interaction between sub-Experiment, Condition, and Congruency was not significant [F(2, 120) = 0.84, *p* =.434].

#### Questionnaire analysis

First, we analyzed the questionnaire data of the experiment that presented real facial stimuli. We recruited Related-Samples Friedman’s Two-Way Analysis of Variance test to analyze valence (between very unpleasant [1] and very pleasant [7]) and arousal (between very calm [1] and very excited [7]) ratings between happy^a^ (1), happy^b^ (2), angry^a^ (3), angry^b^ (4), sad^a^ (5), sad^b^ (6), angular shape (7), and round shape (8). Notice that the superscripts ’a’ and ’b’ refer to the identity of the person in the face stimuli. Concerning valence ratings, the analysis revealed a significant difference in ratings between the variables [CHI^2^(7) = 64.2, *p* <.001]. Table 2 shows the means and SDs for each variable and which variables significantly differ from each other according to ratings.

**Table 2:** The tables present the mean rates and standard deviations of the stimuli used in experiment 2 (the real face sub-Experiment). Notice that the superscripts ’a’ and ’b’ refer to the identity of the person in the face stimuli. The upper table presents the estimated Valence rates, while the lower table presents the estimated arousal rates. The tables also present the outcome of Related-Samples friedman’s Two-Way analysis of variance test. The significant p-values between the evaluations of two stimuli signal that the evaluations differ significantly from each other (ns = not significant)

The estimated valence of the stimuli								
	mean/SD	happy^b^	angry^a^	angry^b^	sad^a^	sad^b^	angular	round
happy^a^	6.6/0.7	ns	< 0.001	< 0.001	< 0.001	< 0.001 **	ns	ns
happy^b^	6.4/0.5		< 0.001	< 0.001	< 0.001	< 0.001 **	ns	ns
angry^a^	2.4/0.9			ns	ns	ns	ns	= 0.016
angry^b^	2.6/1.4				ns	ns	ns	ns
sad^a^	2.5/1.1					ns	ns	ns
sad^b^	2.7/0.9						ns	ns
angular	4.2/1.3							ns
round	5.4/1.7							
The estimated arousal of the stimuli								
	mean/SD	happy^b^	angry^a^	angry^b^	sad^a^	sad^b^	angular	round
happy^a^	3.2/1.1	ns	= 0.005	= 0.009	= 0.005	ns	< 0.001 **	ns
happy^b^	2.5/1.5		< 0.001	< 0.001	< 0.001	= 0.015	< 0.001 **	ns
angry^a^	5.4/1.1			ns	ns	ns	ns	= 0.006**
angry^b^	5.2/0.8				ns	ns	ns	= 0.012 **
sad^a^	5.4/0.7					ns	ns	= 0.006 **
sad^b^	4.3/0.9						ns	ns
angular	5.8/1.1							< 0.001 **
round	2.9/1.3							

Second, we analyzed the questionnaire data of the sub-Experiment that presented emoji facial stimuli. We recruited Related-Samples Friedman’s Two-Way Analysis of Variance test to analyze valence (between very unpleasant [1] and very pleasant [7]) and arousal (between very calm [1] and very excited [7]) ratings between happy (1), angry (2), sad (3), angular shape (4), and round shape (8). Concerning valence ratings, the analysis revealed a significant difference in ratings between the variables [CHI^2^(4) = 31.6, *p* <.001]. Table 3 shows the means and SDs for each variable and which variables significantly differ from each other according to ratings. Concerning arousal ratings, the analysis revealed a significant difference in ratings between the variables [CHI^2^(4) = 36.9, *p* <.001]. 

**Table 3:** The tables present the mean rates and standard deviations of the stimuli used in experiment 2 (emoji sub-Experiment). The upper table presents the estimated Valence rates, while the lower table presents the estimated arousal rates. The tables also present the outcome of Related-Samples friedman’s Two-Way analysis of variance test. The significant p-values between the evaluations of two stimuli signal that the evaluations differ significantly from each other (ns = not significant)

The estimated valence of the stimuli					
	mean/SD	angry	sad	angular	round
happy	6.0/0.6	< 0.001	= 0.006	= 0.029	ns
angry	2.2/1.2		ns	ns	= 0.010
sad	3.2/1.1			ns	ns
angular	3.7/1.6				ns
round	4.7/1.5				
The estimated arousal of the stimuli					
	mean/SD	angry	sad	angular	round
happy	2.6/1.4	= 0.003	ns	< 0.001	ns
angry	5.8/1.1		= 0.016	ns	< 0.001
sad	2.9/1.4			= 0.003	ns
angular	6.2/0.7				< 0.001
round	2.5/1.4				

### Discussion

The results of Experiment 2 showed that angry facial expressions are solely associated with the angular shape, and happy facial expressions are solely associated with the round shape. However, sad facial expressions are associated with angular or round shapes depending on whether they are contrasted with angry or happy faces in a particular block. When they are contrasted with angry faces, they are associated with the round shape; when they are contrasted with happy faces, they are associated with an angular shape. These IAT effects that were observed between shapes and emotions were highly significant, and the effect sizes signaled that the effects were medium or large, suggesting that these interactions are relatively robust. In general, it appears that these shape-emotion correspondences might be more robustly based on an experienced arousal of particular shapes instead of an experienced valence of these shapes. That is because, for example, regarding the shape-emotion correspondence observed with the emoji faces in the sad-angry block, according to the explicit evaluation, the effect seems to be based on associating sad and angry facial expressions with different degrees of arousal instead of valence. This experiment showed that the shape-emotion correspondence can be also observed in implicit association tasks and supports the view that the implicit kiki-bouba effect may be mediated by emotional processes.

## Experiment 3

The results of Experiment 1 did not reveal a significant interaction between the kiki-bouba like words and the aroused-calm facial expressions. One reason for the lack of this effect could be that the task in Experiment 1 emphasized processing the arousal of the emotional stimuli instead of the valence of these stimuli. Hence, Experiment 3 focuses on further researching whether particular emotions are implicitly associated with the kiki- and bouba-like words. This study uses the same facial emoji stimuli as used in Experiment 2. Hence, we assume that if the kiki-bouba effect were based on emotional mediation processes between the shape and the pseudo-word, the pattern of results observed between emotional facial emoji stimuli and the pseudo-word should mirror the pattern of results observed between the emotional emoji stimuli and the shapes in Experiment 2. That is, in the angry-happy condition, the angry facial expression should be associated with the [tɑketɑ] and the happy expression with the [mɑbemɑ], in the angry-sad condition, the angry facial expression should be associated with the [tɑketɑ] and the sad expression with the [mɑbemɑ], while in the sad-happy condition, this sound-emotion correspondence should be absent.

### Method

#### Participants

In Experiment 3, eighteen people participated in the study (19–33 years of age; mean age = 23.1 years; two males; all right-handed). All participants had normal or corrected-to-normal vision and hearing and were native Finnish speakers. All participants were naive to the purpose of the study. The informed consent to participate in the study was obtained from participants. None of the participants took part in multiple experiments. The study was conducted according to the principles expressed in the Declaration of Helsinki. Written informed consent was obtained from all participants, and the participants were able to withdraw their consent and participation at any time without consequence. The participants received gift cards valued at 20 euros as compensation for participating. The study was approved by the Ethical Review Board in the Humanities and Social and Behavioral Sciences at the University of Helsinki.

#### Apparatus, stimuli, procedure, and statistical analyses

The apparatus, procedure, and statistical analyses were the same as in Experiments 1 and 2. In Experiment 3, the face stimuli consisted of the same emoji faces that were used in Experiment 2. The only difference between Experiment 2 and 3 was that in Experiment 3 the angular and round shapes were replaced by the [tɑketɑ] and [mɑbemɑ] words. The exact details of these auditory stimuli are described in the method section of Experiment 1. The questionnaire of Experiment 3 tested the experienced valence (scale between 1 [very unpleasant] and 7 [very pleasant]) and arousal (scale between 1 [very calm] and 7 [very excited]) of the facial expressions (of the emoji faces) as well as the words used in the study. Regarding independent variables, the GLMM analyses of the IAT data treated block Condition (1 = [angry^a^/happy^b^] emotion - [tɑketɑ^c^/mɑbemɑ^d^] word, 2 = [sad^a^/happy_b_] emotion– [tɑketɑ^c^/mɑbemɑ^d^] word, 3 = [angry^a^/sad^b^] emotion– [tɑketɑ^c^/mɑbemɑ^d^] word) and block Congruency (1 = congruent block, 2 = incongruent block) as fixed within factors. Notice that in each block Condition, the item that is signaled by the superscript ’a’ is hypothesized to be congruent with the item that is signaled with the superscript ’c’, while the item that is signaled by the superscript ’b’ is hypothesized to be congruent with the item that is signaled by the superscript ’d’.

### Results

**Fig. 3:**
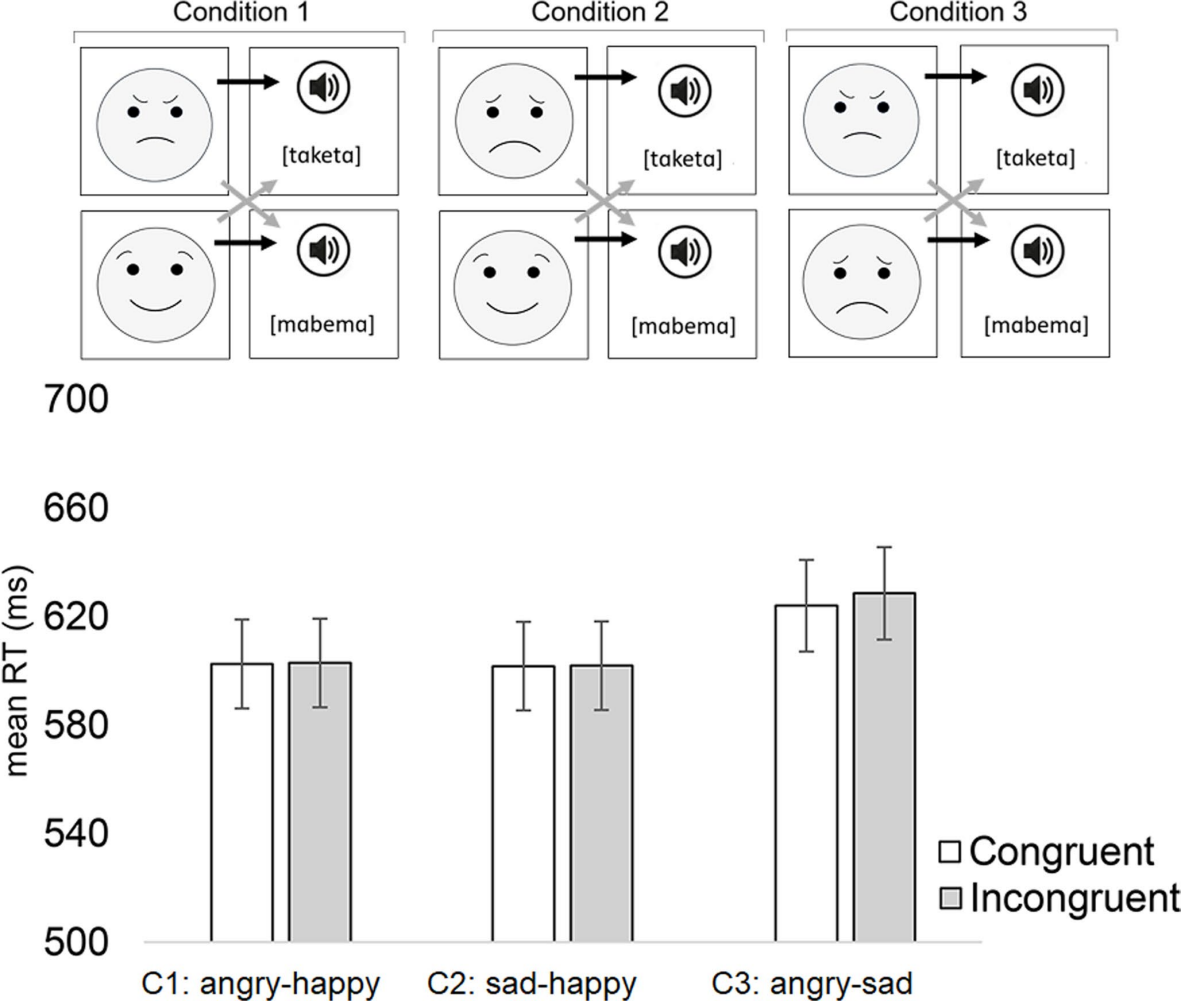
(Above) The illustration of the stimuli and design used in Experiment 3. The black arrows refer to congruent stimulus pairs whereas grey arrows refer to incongruent stimulus pairs. (Below) The mean manual reaction times for Experiment 3 as a function of the congruency for the conditions of angry-happy, sad-happy, and angry-sad. In the angry-happy condition, the congruent pairs were angry-[tɑketɑ] and happy-[mɑbemɑ], in the sad-happy condition, the congruent pairs were sad-[tɑketɑ] and happy-[mɑbemɑ], and in the angry-sad condition, the congruent pairs were angry-[tɑketɑ] and sad-[mɑbemɑ]. Error bars depict the standard error of the mean

*Reaction times*: Before analyzing reaction times, all practice trials and erroneous data (i.e., the participant responded with the wrong key or did not produce any response; 4.9%) were removed. Additionally, reaction time data more than 2 standard deviations from the mean (performed individually for the data of each participant) were excluded (4.6%). The analysis did not reveal a significant main effect of Congruency [F(1, 9390) = 0.09, *p* =.766] or interaction between Condition and Congruency [F(2, 9390) = 0.19, *p* =.826]. The mean reaction times and pairwise contrasts of this interaction are provided in Fig. 3. Only the main effect of Condition was significant [F(2, 9390) = 6.45, *p* =.002] (Condition 1: M = 603 ms; Condition 2: M = 602 ms; Condition 3 = 626 ms). The results of erroneous responses did not reveal any significant effects (Condition: [F(2, 93) = 0.05, *p* =.950]; Congruency: [F(1, 93) = 0.01, *p* =.952]; Condition*Congruency [F(2, 93) = 0.18, *p* =.839]).

#### Questionnaire analysis

**Fig. 4:**
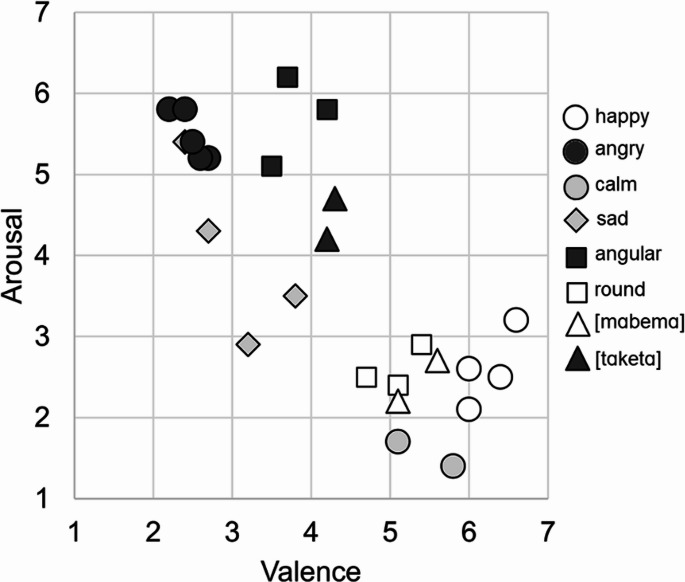
The mean estimated valences and arousal values of stimuli used in Experiments 1–3 across the emotional dimensions of valence and arousal

We recruited Related-Samples Friedman’s Two-Way Analysis of Variance test to analyze valence (between very unpleasant [1] and very pleasant [7]) and arousal (between very calm [1] and very excited [7]) ratings between happy-emoji (1), angry-emoji (2), sad-emoji (3), taketa sound (4), and mabema sound (5). Concerning valence ratings, the analysis revealed a significant difference in ratings between the variables [CHI^2^(4) = 50.4, *p* <.001]. Table 4 shows the means and SDs for each variable and which variables significantly differ from each other according to ratings. Concerning arousal ratings, the analysis revealed a significant difference in ratings between the variables [CHI^2^(4) = 43.5, *p* <.001]. Figure 4 presents the mean estimated valences and arousal values of stimuli used in Experiments 1–3 across the emotional dimensions of valence and arousal.

**Table 4:** The tables present the mean rates and standard deviations of the stimuli used in experiment 3. The upper presents the estimated Valence rates, while lower presents the estimated arousal rates. The tables also present the outcome of Related-Samples friedman’s Two-Way analysis of variance test. The significant p-values between the evaluations of two stimuli signal that the evaluations differ significantly from each other (ns = not significant)

The estimated valence of the stimuli					
	mean/SD	angry	sad	[tɑketɑ]	[mɑbemɑ]
happy	6.0/0.8	< 0.001	< 0.001	= 0.013	ns
angry	2.4/1.1		ns	= 0.044	< 0.001
sad	3.8/0.9			ns	= 0.022
[tɑketɑ]	4.3/1.4				ns
[mɑbemɑ]	5.6/1.0				
The estimated arousal of the stimuli					
	mean/SD	angry	sad	[tɑketɑ]	[mɑbemɑ]
happy	2.1/1.1	< 0.001	ns	< 0.001	ns
angry	5.8/1.2		= 0.011	ns	< 0.001
sad	3.5/1.0			ns	ns
[tɑketɑ]	4.7/1.3				ns
[mɑbemɑ]	2.7/1.4				

### Discussion

Most importantly, contrary to the shape-emotion correspondence observed in Experiments 1 and 2, the results of Experiment 3 replicated the absence of the sound-emotion correspondence observed in Experiment 1. The results of the IAT did not reveal any association between the kiki/bouba-like words and facial expressions. None of the interaction effects between pseudowords and facial expressions were even close to being statistically significant. Hence, it seems that people do not automatically process any emotional dimensions of the perceived kiki/bouba-like words when the task is to discriminate a kiki-like word from a bouba-like word. However, similar to the results of Experiment 1 in which the explicit evaluation task associated the angry facial expressions with the [tɑketɑ] and the calm facial expressions with [mɑbemɑ] in terms of arousal rates, in Experiment 3, the angry facial expression was associated with the [tɑketɑ] and the happy facial expression with the [mɑbemɑ] in terms of arousal rates. These observations replicate the results reported by Aryani et al. (2020) showing that kiki-like words are associated with high-arousal stimuli, whereas bouba-like words are associated with low-arousal stimuli in the tasks that require an explicit emotional evaluation of the stimuli.

## General discussion

Experiment 1 replicated the “explicit” kiki-bouba effect (Köhler, 1929) (i.e., the effect based on associating explicitly the shapes and pseudowords). That is, the angular shape was explicitly linked to the word [tɑketɑ], and the round shape was linked to the word [mɑbemɑ]. In addition, similarly to the previous studies (Chuquichambi et al., 2022; Aryani et al., 2020), the angular shape was experienced as more aroused than the round shape and the word [mɑbemɑ]. Valence rates did not significantly differentiate the shapes and the kiki/bouba-like words. These findings support the view that the kiki-bouba effect, at least the explicit version of the effect, might be mediated by the higher-order factor of arousal associated with the angular shapes and kiki-like words (Aryani et al., 2020).

 Given that one of the primary goals of this study was to investigate whether the kiki-bouba effect would be based on implicitly operating emotion-related mediation processes, in Experiment 1, the kiki-bouba effect was also observed in the implicit association test (IAT), replicating the results observed by Parise and Spence (2012). It is noteworthy that the kiki-bouba effect, linked to the explicit and implicit association tests, was similarly observed with participants who knew the phenomenon beforehand and who were not familiar with the phenomenon, albeit the effects were somewhat more robust for those who were familiar with the effect. However, in contrast to the observed associations between shapes and facial expressions, in the IAT of Experiment 1, the [tɑketɑ]/[mɑbemɑ] words did not reveal associations with emotional facial expressions. That is, the angular shape was associated with the angry facial expression, while the round shape was associated with the calm facial expression. In contrast, the [tɑketɑ]/[mɑbemɑ] words were not associated with these facial expressions when the task emphasized implicit association processes. These observations were replicated in Experiments 2 and 3, in which the shape was associated in the IAT with specific facial expressions (Experiment 2), while the kiki-bouba-like words were not associated with these same emotional facial expressions (Experiment 3). These observations suggest that, in the IAT, participants might not process the emotional dimension of the perceived kiki-bouba-like words to the degree that would be sufficient for mediating the association between the shape and the word. It is not likely that people would process the potential emotional dimensions of the perceived kiki/bouba-like words when implicitly associating shapes with pseudo-words (in Condition 3 of Experiment 1) because they do not process this emotional information of these words even when the task requires that half of the stimuli presented in a given block are discriminated based on their emotional content (Condition 1 of Experiment 1).

The “implicit” kiki-bouba effect (i.e., the effect observed in an implicit association task) appears to be based on other cognitive mechanisms than emotional mediation, such as automatic simulation of the perceived shape and pseudo-word in articulatory processes, and/or associating learned representations of sounds that different shapes naturally produce with learned representations of acoustic consequences of pronounced kiki and bouba-like words. It is also possible that all these higher-order elements of shapes and sounds– emotional, articulatory, and acoustic– are potentially available for associating a particular shape with a particular speech sound, and that the task determines which one of these higher-order properties of represented shapes and pseudo-words mediate the association process. Perhaps, explicit association tasks stress emotional mediation processes, while implicit association tasks stress, for example, acoustic and articulatory processes. In line with this view, the recent neural and cognitive models of semantic representation assume that conceptual knowledge of objects and events integrates modality-specific information related to the referent object/event - perceptual, affective, and motor information in particular - and abstracts this integrated information at the supramodal level (e.g., Patterson et al., 2007; Binder & Desai, 2011). If applying these models to explain the kiki-bouba effect, for example, kiki-like words can be associated with sharp shapes because conceptual representations of these words and shapes share modality-specific knowledge in terms of acoustic, motor, and emotional processes. The ongoing task might specify whether and to what extent cognitive processing recruits all or some of these modality-specific components when the objects are perceived, represented at the semantic level, and associated with each other.

The study also provided further evidence for the shape-emotion correspondence. Previous studies that have investigated shape-emotion correspondence have mostly used tasks in which participants are required to explicitly judge the emotional values of angular and round shapes. Most typically, these studies have revealed that round shapes are experienced as more pleasurable than angular shapes (Chuquichambi et al., 2022). The results of the present study somewhat replicated these observations. In the explicit emotion evaluation task, the angular shape was associated with the angry facial expressions, while the round shape was associated with the calm facial expressions. In addition, the present study showed that these interactions can also be observed in the IAT when participants’ responses are based on discriminating the angular shape from the round shape, rather than an explicit evaluation of the emotional dimension of perceived shapes. In Experiments 1 and 2, the sharp shape was implicitly associated with the angry faces, and the round shape was associated with the happy and calm faces. If the emotional basis of these interaction effects is considered according to the explicit emotion evaluation of different facial expressions, it seems that these shape-emotion correspondences might be more robustly based on an experienced arousal of particular shapes instead of an experienced valence of these shapes. Particularly in Experiment 1, the emotional evaluations of the calm facial expressions, which were associated with the round shape in the explicit and implicit tasks, differed from the emotional evaluations of the angular shape in terms of arousal rates but not in terms of valence rates.

Interestingly, regarding the sad faces of real people in Experiment 2, the shape-emotion interaction of the IAT depended on whether the face was paired with the angry or happy face. When it was paired with a happy face, it was associated with the angular shape, and when it was paired with an angry face, it was associated with the round shape. Regarding the shape-emotion correspondence observed with the faces of real people in the happy-sad block, the explicit emotion estimates propose that these shape-emotion correspondences were based on experienced arousal instead of experienced valence of angular and round shapes. That is because the evaluations of the happy expressions differed from those of the angular shape, and the evaluations of the sad expressions differed from those of the round shape, primarily in terms of the arousal rates instead of the valence rates. Regarding the shape-emotion correspondence observed with the emoji faces in the sad-angry block, this effect seems to again be based on associating sad and angry facial expressions with different degrees of arousal instead of valence. The emotion evaluations of the sad expression differed from those of the angular shape, and the emotion evaluations of the angry expression differed from those of the round shape more robustly in terms of arousal rates than valence rates. Correspondingly, the shape-emotion correspondence was not observed with the emoji faces in the happy-sad block, perhaps for the reason that the explicit emotion evaluations of the sad and happy expressions similarly differed from the explicit emotion evaluations of the angular shape in terms of arousal. That is, it appears that the shape-emotion correspondence is missing if the two emotions that are included in the shape-emotion block do not differ in terms of experienced arousal.

Concerning sound-emotion correspondence, previous investigations reveal that kiki-like words are explicitly associated with higher arousal values than bouba-like words (Aryani et al., 2020). This observation was also replicated in the present study. The explicit emotion evaluation task of Experiment 1 showed that the angry expression was associated more strongly with the [tɑketɑ], while the calm expression was associated more strongly with the [mɑbemɑ]. The results of the explicit emotion evaluation task do not conclusively reveal whether this sound-emotion effect is based more on arousal or valence. The angry facial expressions differ from the [mɑbemɑ] and the calm facial expressions differ from the [tɑketɑ] in terms of valence rates as well as arousal rates, although it seems that the calm facial expressions differ from the [taketa] more robustly in terms of arousal rates than valence rates. However, even though the explicit arousal evaluations of Experiments 1 and 3 suggest that anger is associated with kiki-like words and calmness as well as happiness with bouba-like words, these sound-emotion correspondences were missing in the implicit association tasks of Experiment 1 and 3. This observation suggests that people do not automatically process the emotional dimensions of kiki and bouba-like words when responses are based on discriminating a kiki-like word from a bouba-like word (e.g., implicit association tests). As a consequence, it is not likely that the implicit kiki-bouba effect would be based on emotional mediation processes.

It has been shown that emotional dimensions can be extracted from phonemes even when the experimental task does not require an explicit processing of these emotional dimensions. Schmidtke et al. (2024) showed that perceiving positive facial expressions automatically facilitates responding to pseudo-words containing the vowel /i/ (e.g., Zinal), whereas perceiving negative facial expressions facilitates responding to pseudo-words containing the vowel /o/ (e.g., Zonal) supporting the view that the front vowel /i/ is associated with positive valence, while the back vowel /o/ is associated with negative valence (Körner & Rummer, 2022). Although the present study (Experiment 1) showed that people explicitly associate the angry expression with the [tɑketɑ] and the calm expression with the [mɑbemɑ], this effect was not observed in the implicit association task. Perhaps, higher-order emotional connotations are more tightly anchored in vowels than in consonants. As a consequence, it is possible because the present study only manipulated the consonant content of the pseudo-words, the sound-emotion correspondence effect was only observed in the explicit task. Therefore, it is possible that we would have observed the implicit sound-emotion correspondences if we would have manipulated the vowel contents of kiki/bouba-like words instead of solely manipulating the consonant content of these words.

### Potential limitations of the study

One of the potential limitations of the study was that even though the study would have revealed the sound-emotion correspondence in the implicit association test in the same way as the correspondence was observed between shapes and emotions, this would not have conclusively established that angular/round shapes are associated with kiki/bouba-like words due to emotional mediation processes. However, the fact that emotional stimulus dimensions were implicitly processed in relation to perceived shapes but were not implicitly processed in relation to perceived kiki-bouba-like words suggests that, at least, the implicit kiki-bouba effect observed in Experiment 1 is not based on emotional mediation processes.

Another potential limitation of the study was that the sample size was smallish in the present study. Increasing the sample size for future studies might enhance the generalizability of the findings. Moreover, although the kiki-bouba effect has been observed in most of the cultures and language environments (see, for example, Bremner et al., 2013), some failures to observe the effect have also been reported, such as in the Songe of Papua New Guinea (Rogers & Ross, 1975). Therefore, the generalizability of the present findings could be researched by increasing the sample size as well as exploring the effects observed in this study in different language environments.

In addition, it is noteworthy that we used only two pseudo-words in the study, and only the consonant content of these words was manipulated. Instead, for example, Aryani et al. (2020) used a large number of different pseudo-words in their study, and they also manipulated the vowel content of these words in addition to the consonant content. We used only two pseudo-words to keep the length of the experiment reasonable for the participants, and only the consonant content was manipulated because it has been shown that consonants play a key role in the kiki-bouba effect (Nielsen & Rendall, 2011; Fort et al., 2015). Moreover, it has to be emphasized that, in the present study, the kiki-bouba effect was robustly observed in the implicit and explicit association tasks showing that if the observed kiki-bouba effect was mediated by emotional processes, the emotional dimensions of these particular words should have been processed to the extent that the implicit sound-emotion correspondence effect would have been observed. That is, in order to accept that the kiki-bouba effect observed in the implicit association task with these particular words and shapes was based on emotional mediation processes, the [tɑketɑ]/[mɑbemɑ] words should have been implicitly associated with the same emotions as the angular/round shapes, respectively. This prerequisite was clearly not achieved in the study.

Recent research suggests that when exploring implicitly operating associations between valence representations, the BIAT (brief implicit association test) effect is sensitive to semantic (nonexperiential) instead of affective (experiential) representations of valence (Argaman et al., 2024). Therefore, in the present study, the participants could encode the emotional facial expression and shapes in a nonexperiental (conceptual) format (e.g., negative vs. positive). If this is so, it is possible that the IAT is not a sufficiently sensitive method to investigate emotional connotations linked to pseudowords and shapes. In that case, the present findings could be interpreted to suggest that in order to enable emotional mediation of the kiki-bouba effect, the task should emphasize representing the shapes and pseudowords at the experiential (affective) level. This level of processing might take place, for example, when the task requires explicitly associating the shapes with pseudowords.

Furthermore, given that, in the present study, the task was to categorize facial expressions (e.g., angry vs. happy), the arousal dimension was only embedded in the stimuli. However, the previous research suggests that only the relevant emotional dimension can have an impact on the IAT effect (De Houwer, 2001). Hence, although the explicit evaluation tasks of the study suggest that the correspondence effects observed in the IAT were likely to be based on the arousal dimension of the stimuli, this might be an overinterpretation of the results. In general, it might be reasonable to question how well the outcomes of the explicit emotion evaluation tasks can be applied to interpret the results of the IAT. As such, the emotion-related IAT effects of the present study may also reflect representing the perceived stimuli at the level of negative/positive concepts. Hence, in future studies, it might be important to investigate whether similar emotion-related correspondence effects to those observed in the present study could also be observed if the facial stimuli were replaced by stimuli that are more straightforwardly represented within positive-negative axis (e.g., words having negative/positive connotations, such as *murder* vs. *love*).

In conclusion, the study shows that the implicit kiki-bouba effect is not likely to be based on emotional mediation processes. We suggest that, at least, the implicit kiki-bouba effect is based on implicit simulation of the perceived pseudo-word and shape in articulatory processes and/or implicitly linking learned representations of acoustic consequences of pronounced kiki and bouba-like words to learned representations of sounds that different shapes naturally produce. Regarding the shape-emotion correspondences, the study showed that this phenomenon can be robustly observed in explicit as well as implicit association tasks and that it might be more robustly based on arousal instead of valence of perceived shapes. Finally, the sound-emotion correspondence was only observed in the task that required explicit mapping between pseudo-words and emotions. The explicit emotion evaluation did not provide clear results on whether this effect was based on experienced arousal or valence of these pseudo-words. However, the study showed that this sound-emotion phenomenon is absent in the implicit association task, suggesting that people do not automatically extract emotional information from perceived phonemes. Regarding future investigations, research should test whether similar outcomes to those observed in the present study would be observed, for example, if the facial stimuli were replaced by stimuli that are more straightforwardly represented within positive-negative axis, and if the kiki/bouba-like stimuli would include wider variety of different kinds of pseudowords that are presented visually and aurally.

## Data Availability

The research data is submitted to the following repository: https://osf.io/5eqkg/?view_only=19eee5cb010f42479a30e2c06844c664.
